# Experiences of autistic women in menopause: brief review and recommendations for practice and research

**DOI:** 10.3389/frph.2026.1762773

**Published:** 2026-02-19

**Authors:** Natalie M. Badgett, Lisa Taylor-Swanson, Stephanie Quist, Jane Price, Jamie Villanueva

**Affiliations:** 1 Department of Special Education, University of Utah, Salt Lake, UT, United States; 2 College of Nursing, University of Utah, Salt Lake, UT, United States; 3 Martha Bradley Evans Center for Teaching Excellence, University of Utah, Salt Lake, UT, United States

**Keywords:** autism, autistic women, brief review, interoceptive awareness, menopause

## Abstract

Autistic women navigating the menopause transition face a constellation of challenges that remain critically understudied. This paper explores the intersection of autism-specific traits and menopausal symptoms, with a focus on interoceptive awareness (IA)—the ability to perceive internal bodily signals—which is frequently dysregulated in autistic individuals. Dysregulated IA may contribute to misinterpretation of menopausal symptoms, which in turn amplifies vasomotor severity, anxiety, depression, and distress during this life stage. Systemic barriers further complicate care access. Autistic women often encounter communication challenges with healthcare providers, limited provider knowledge of autism and menopause, and reduced social support. Addressing these gaps requires interdisciplinary approaches, including autism-informed health education, clinician training, IA-targeted interventions, and peer support networks. This paper calls for expanded research into the relationship between autism, interoception, and menopause to inform clinical practice and improve quality of life for autistic women during midlife transitions.

## 1 Introduction

Over the past decade, autism diagnoses have increased more than threefold among women and girls, potentially indicating heightened awareness of autism in females (1). Despite these findings, a significant gap exists in research exploring the unique health considerations of autistic women, particularly regarding the menopausal transition (2, 3). This issue is further compounded by the challenges women face in accessing menopause education and support (4). As autism rates continue to rise, there is a growing need for research that specifically examines the experiences and health concerns of autistic women throughout menopause (3). The purpose of this brief review is to provide an overview of current research concerning autistic experiences during the menopausal transition and post menopause. In doing so, we will provide a summary of autism and the specific experience of autism among women, including common co-occurring physical, behavioral, and mental health issues that likely impact the experiences of menopause in this population. Finally, we will suggest avenues for future interdisciplinary research to better understand lived experience and potential supports.

## 2 Autism and menopause

### 2.1 Autism

Autism is a neurodevelopmental disorder defined by core characteristics related to deficits in social behavior and communication, occurring in 1 in 31 children in the United States according to the most recent prevalence data (5). Autism is typically diagnosed in early childhood (i.e., age 5) (6) can be diagnosed in adulthood when there is a documented history of symptoms in childhood (7). Health care database evidence (i.e., insurance claims) suggests that increases in autism diagnosis rates between 2011 and 2022 were greatest among adults, particularly females (1). Historically thought to occur primarily in males, recent research suggests that autism in females may be underdiagnosed or misdiagnosed, and calls have emerged in the past decade for research to attend to gender differences in the autistic experience (8). Females with autism are diagnosed significantly later than males, especially when they do not experience a co-occurring intellectual disability (9, 10). Because autism is a developmental disorder, symptoms sustain through adolescence and adulthood for most individuals with the diagnosis. Severity of symptoms varies widely across individuals diagnosed with autism, defined in diagnosis based on the level of support needs that an individual might require (11).

Beyond core characteristics of autism, co-occurring mental health (e.g., anxiety, depression, obsessive compulsive disorder) and physical (e.g., gastrointestinal issues, disordered sleep, epilepsy) conditions occur at significantly higher rates among autistic individuals than in the general population (12, 13). As inclusion of autistic females in research has increased with increases in their diagnosis, research suggests that may be more likely to experience co-occurring mental health conditions. For example, depression and anxiety appear to present in autistic females from childhood through adulthood, and slightly higher risk of suicide among autistic females has been raised as a concern in recent research (14). Additionally, behavioral health concerns are common among individuals with autism. Current research suggests that up to 90% of children with autism engage in some form of interfering behavior (i.e., behavior that is persistent, destructive, harmful, and limits access to educational and community environments) (15). While some research suggests that the prevalence of challenging behavior declines as individuals age past young adulthood, prevalence in adulthood appears to be related to autism symptom severity (e.g., language impairment) as well as the presence of some co-occurring health diagnoses (e.g., disordered sleep, gastrointestinal issues) (16, 17).

Interoceptive awareness (IA) refers to the ability to perceive and interpret internal bodily sensations, such as hunger, pain, and thirst (18). Individuals diagnosed with autism may have heightened IA, decreased IA, or perceive that something is wrong internally but are unable to locate the area of discomfort or pain (19). A recent meta-analysis (20) investigating differences in IA between autistic individuals and neurotypical individuals found evidence suggesting that people with autism experience difficulties in IA to a greater extent than the neurotypical population. While these difficulties are neither prevalent nor extensive enough to represent a core symptom of autism, they do hold implications for emotional regulation and physical wellbeing (21). For instance, an interoceptive-focused intervention decreased trait anxiety in autistic adults and improved interoceptive accuracy, as measured by a heartbeat detection task—potentially supporting move effective self-regulation strategies (21, 22). The extent to which an individual with autism experiences mental, behavioral, and physical health concerns in addition to autism, as well as difficulties related to IA, will impact their access to community and educational supports, including health care.

#### 2.1.1 Health care needs and experiences among individuals with autism

Individuals with autism experience unique challenges related to accessing high quality health care. These challenges include increased barriers to accessing providers, higher unmet needs, and lower satisfaction with care than individuals without autism (23, 24). Current research suggests that health care experiences among individuals with autism are impacted by the complexity of their physical and mental health care needs (25) as well as a lack of training among primary and specialty health care providers related to autism (26, 27). As with other populations, difficulties in accessing health care increase as needs become more complex.

Health care needs among individuals with autism are uniquely exacerbated among autistic women, largely due to the lack of existing research on the experiences of women with autism. Due to the higher prevalence of autism diagnoses in men than in women, autism research has primarily focused on symptom presentation and experiences among autistic men and boys (28). Current research suggests that women have been historically misdiagnosed or received their diagnosis later in life than men due to a lack of understanding and consensus on the presentation of autism among women (28). Women without co-occurring intellectual disability are especially at risk for missed or late diagnosis; Zener (29) described that common paths to diagnosis for this population include seeking diagnosis after the diagnosis of a family member (i.e., their child), identifying traits in other autistic women, and coming to the diagnosis as they are seeking support for mental health and burnout (29).

#### 2.1.2 Experiences of autistic women

Traditionally thought to occur predominantly in males (30–32) current research suggests that autism occurs in females more often than previously thought and that the disorder may present differently in affected females (33, 34). Due to the lack of existing research focused on autistic women, little is known about health care needs specific to this population. As the prevalence of women diagnosed with autism increases, research is needed to better understand symptomology, lived experiences, and intervention approaches so that providers are equipped to deliver effective care for health issues specific to autistic women. Specifically, research is needed to support autistic women as they experience health care needs associated with physical experiences unique to females, including menstruation, pregnancy and childbirth, and menopause (14). These conditions, common among females, present complex challenges for physical and mental health that are understudied among the autistic population.

### 2.2 Menopause

Menopause is the transition from the reproductive to the post-reproductive stage of life experienced by nearly all women, characterized by cessation of menstrual cycles. Many women experience accompanying physical and mental health changes related to hormonal changes during this transition (35). An estimated 85% of women experience vasomotor symptoms (36) such as hot flashes, night sweats, and palpitations for up to a decade (37) during peri- and post-menopause. Women may also experience pain, problems with sleep or cognition, anxiety, and depression during menopause (38). These symptoms lead to a decreased menopause-related quality of life (39) and reduced productivity (40), costing the US healthcare system an estimated $24.8 billion annually (41). The majority of women do not receive adequate education or care for menopause related symptoms (42).

#### 2.2.1 Health care for women experiencing menopause

Many women do not receive adequate education or care for menopause related symptoms (42), resulting in limited knowledge about the transition and ultimately leaving them unprepared to manage associated physical and psychological changes. Existing research indicates that over 80% of women report receiving no formal menopause education and that mid-life women rely primarily on informal sources like websites and friends for information (43). When women do try to receive medical care, they often report being left untreated (42). One recent study of health records revealed that 40% of women included in the study did not receive a prescription for menopausal symptoms, and 13% received no treatment at all (42). Further exacerbating the lack of support, only about one-third of OB/GYNs report receiving training in menopause management during their residency (44).

#### 2.2.2 Autistic experiences in menopause

For autistic women, menopause may be especially challenging given the defining characteristics of autism; communication difficulties may further impede access to high quality medical care for menopause related issues, social challenges may impact access to support systems demonstrated as important for menopausal women (11), and sensory processing issues that commonly co-occur with autism and further increase challenges related to interoceptive awareness that is common both for individuals with autism and in women experiencing menopause (45). Specifically, autistic women experiencing perimenopause and menopause are likely unable to identify the source of their symptoms, and their ability to explain their physical sensations may be further exacerbated by difficulties in communication as well as challenges in accessing high-quality health care generally (2).

Existing research examining the needs and experiences of autistic women is limited, particularly in health care issues specific to women's health. Menopause is a transitional health experience shared by nearly all women, and yet little is known about the needs of autistic women navigating menopause. While further research is needed, the limited existing research provides some understanding of lived experiences, symptomology, and current interventions; each of these areas of research is summarized below and should inform future research and approaches to care.

#### 2.2.3 Presentation of menopause Among autistic women

The presentation of menopausal symptoms among autistic women appears to be more intense and complex than what is experienced by their neurotypical counterparts (46). Symptoms reported often include hot flashes, amplified sensory sensitivities (i.e., atypical sensory experience (47), increased emotional volatility, behavioral challenges, sleep challenges, and cognitive impairments related to focus, memory, and decision making (45, 48, 49). Often, a regression or intensification of common autistic characteristics associated with IA (e.g., intolerance to change and heightened sensitivity to sensory input) accompanied these symptoms, further exacerbating daily functioning during menopause (45, 48, 49). Rynkiewicz et al. (50) provide an illustrative example of the unique challenges of menopausal autistic women in their case study of a 53-year-old, white, autistic woman who participated in a pilot trial of a multi-component telehealth intervention. At the beginning of the intervention, the participant reported experiencing significantly higher levels of sensory sensitivity and more sensory avoidance compared to general population norms, as measured by the Adult/Adolescent Sensory Profile (AASP). Menopausal Specific Quality of Life Questionnaire scores revealed that overall menopause symptom scores were moderately to severely bothersome, with vasomotor, psychological, and physical symptoms rated as most bothersome. A combination of Hormone Replacement Therapy (HRT), mindfulness meditation, and treatment for a previously undiagnosed pituitary disorder significantly improved vasomotor symptoms, with more limited improvement in psychological and sexual domains (50).

Women with autism describe common menopausal symptoms as particularly disruptive due to pre-existing sensory and regulatory challenges (2). Menopausal symptoms often mirror, overlap with, or intensify existing autism symptoms, characteristics, and co-occurring diagnoses, making it difficult for individuals to distinguish whether their experiences were related to autism or menopause (2). This difficulty is compounded by a widespread lack of understanding within the healthcare community regarding the interplay between autism and menopause (2). Hormonal fluctuations associated with menopause frequently lead to unpredictable physical, mental, and emotional states, contributing to confusion about the origin of these changes and increasing daily distress (49).

Emergent physical health difficulties during this time include fatigue and hot flashes, which are frequently caused by the hormonal imbalances characteristic of the menopausal transition (51). Midlife women also commonly report sleep disturbance, which is associated with both menopause and an autism diagnosis (2). The interplay of hormonal imbalances and neurodivergence, when layered with sensory sensitivities and communication difficulties, increases challenges associated with seeking high-quality care and indicates a need for interdisciplinary care models (e.g., Medical Home) for supporting autistic women in transition (45).

Menopause exacerbates mental health challenges commonly experienced by autistic individuals, including anxiety and depression, which are reported to co-occur at higher prevalence in autistic populations than in neurotypical populations and appear to be especially common in autistic women (8). Ongoing mental health symptoms are heightened during menopause and are often marked by a notable decline in overall mental well-being (52). This decline can be linked to both internal factors, such as hormone-driven mood instability, and external stressors, including inadequate peer and medical support (48). Mental health difficulties during menopause are further intensified by challenges in articulating internal experiences, particularly among individuals who lack a robust emotional vocabulary, are non-speaking, or were undiagnosed when perimenopausal symptoms began (45). Current literature suggests that building a framework to help individuals understand the intersectionality of autism and menopause would be beneficial in contextualizing both lifelong struggles and current levels of distress (2). The formal guidance of a medical practitioner, coupled with peer support, may offer meaningful relief and validation for this population.

Menopause also significantly affects behavioral health among this population, particularly in relation to executive functioning, daily regulation, and routine (2). In addition to exacerbating existing challenging behaviors, menopause may also lead to increased difficulties with time management, task initiation and completion, social interaction, and flexibility among autistic women (45, 53). The addition of menopause to an already dysregulated baseline often results in a regression of functioning (2). For example, one participant in a qualitative study reflected on her experience of menopause: “It seems like we ignored a lot of our traits or just pushed through and didn't really recognize them…emotional regulation has always been an issue…just feels like everything is amplified now” (49). Hormonal fluctuations and intensified disruptions in emotional control contribute to behaviors being misinterpreted or pathologized by a medical community that lacks autism-informed training (54) and approaches specific to this life stage. It is imperative that these behaviors be understood as potentially heightened expressions of existing traits, rather than newly emergent issues. Highlighting the manifestation of behavioral dysregulation because of the distress and physiological imbalance associated with the menopausal transition is essential, as these elements are significantly intertwined and must be interpreted accordingly. For the medical community to respond effectively, strategies and supports must be developed to address the intersectional and interdisciplinary needs of this population.

#### 2.2.4 Access to health care among autistic women experiencing menopause

Across multiple studies (2, 45, 48), autistic females describe the menopausal transition as a particularly isolating and disorienting experience. Their symptom experiences of menopause are often intensified by preexisting difficulties related to sensory processing and IA, executive functioning, and emotion regulation. A recurring theme among individuals seeking help with the menopausal transition was the lack of accessible, autism-informed educational and healthcare resources (2, 45). The convergence of lifelong experiences with autism-related symptoms, whether formally diagnosed or not, further compounded the confusion and distress surrounding the menopausal transition. The masking of symptoms, along with the lack of healthcare and educational approaches informed by the experiences of autistic females, resulted in many women feeling dismissed or misunderstood by the healthcare community. Multiple studies (2, 55) emphasized the profound sense of invisibility felt by these individuals, which was underscored by how the silence around autistic menopausal experiences has been historically reinforced by a lack of research and understanding.

## 3 Discussion

While existing literature examining the experiences of females with autism is limited, the current state of knowledge on their experience with menopause indicates important implications for future practice and research.

### 3.1 Implications for practice

The overlapping symptomatology of autism and menopause underscores the need for an interdisciplinary approach to intervention; when healthcare providers fail to consider the autism-related components of the menopausal transition, they risk misdiagnosing or downplaying the severity and complexity of symptoms in this population. This ultimately compromises not only menopause-related healthcare needs but all healthcare needs of the individual. As highlighted in the sparse existing literature, the healthcare profession must adopt an interdisciplinary framework that is attuned to the unique needs of autistic females and how menopausal symptoms can influence autism and vice versa.

Increasing awareness among healthcare professionals about the intersection of autism and menopause is essential. Many healthcare providers may not be aware of how autism-related traits can overlap and interact with menopausal symptoms. Training programs aimed at educating healthcare professionals in these topics could help them better understand and support autistic patients during this life transition. Additionally, providing support and interventions to improve interoceptive awareness and the interpretation of bodily signals is important due to the associations between autism, alexithymia, and emotional regulation (56). Although the current literature is inconclusive regarding the relationship between autism and interoception, individuals who identify as autistic often report difficulties with interoception (57)., which is the ability to recognize and interpret internal bodily cues (58). Addressing this gap could help individuals better manage their menopausal symptoms, as recent studies have found promise in mindfulness or interoceptive-focused interventions for improving self-regulation and mental health in autistic individuals (22, 59).

Clinicians who are attuned to hallmark features of autism, such as sensory sensitivities, cognitive inflexibility, communication difficulties, and challenges with emotion regulation, will be better equipped to support this population effectively. Given the external factors that shape an individual's day-to-day experience of menopause, providers must also be able to identify and address masking behaviors, the downplaying of symptoms, limited communication abilities, and the obscured interaction between autism and menopause. Integrated care that includes behavioral health professionals, occupational therapists, psychologists, speech-language pathologists, and others who specialize in working with the autistic community—as well as experts in domains impacting menopause symptoms, including IA—is essential for providing comprehensive, informed support.

It is crucial to develop accessible health education about menopause that is specifically tailored for autistic individuals. This would ensure that autistic females experiencing this life transition are informed about what to expect, providing them with resources that are both understandable and sensitive to their needs. Offering alternative communication methods for discussing symptoms with healthcare providers must also be part of this framework. Many autistic individuals face challenges with communication, especially when conveying complex emotions or physical sensations. Providing alternative communication methods—such as visual supports, augmentative and alternative communication (AAC) devices, or even caregiver-assisted communication—can empower autistic women to express their symptoms more effectively. Finally, encouraging the development of peer support networks for autistic women experiencing menopause is vital. Peer support can be invaluable in helping autistic women share experiences, coping strategies, and resources. By connecting women with others navigating similar experiences, these networks can reduce feelings of isolation and provide essential emotional support.

### 3.2 Implications for research

The existing research related to the intersection of autism and menopause is limited. Future research should continue to explore the diverse experiences of autistic females experiencing peri-menopause and menopause, as well as the experiences of health care professionals who provide relevant medical support and intervention for this population. Pursuing such avenues of qualitative inquiry will inform future education, support, and service delivery models to address the needs of this unique patient population by identifying population-specific barriers and facilitators to high quality menopause supports. In addition to conducting exploratory research to understand the needs and experiences of both patients and providers, strategies for menopause support that are tailored to the health, social, and communication needs of autistic women must be developed and evaluated. Furthermore, it will be essential to include autistic females in the development and evaluation of such strategies to ensure relevance to their unique menopausal experiences and health care priorities. Finally, scaling effective strategies for menopause care and support will be necessary and may include pre-service training for healthcare providers in topics relevant to menopause and autism (e.g., IA, communication, and social needs), as well as the development of packaged adaptations to existing support models and approaches for females experiencing menopause.

## 4 Conclusion

As the autistic population ages, and as researchers continue to learn more about the presentation of autism in the female population, it will be imperative to support autistic females in their experience of menopause. While limited, existing literature suggests that females with autism experience unique symptoms of menopause, potentially due to overlapping symptoms that exacerbate challenges faced in the broader population of women in perimenopause and menopause. As such, future research should focus on improving understanding of this unique population, training health care providers to implement high quality and interdisciplinary care and developing and scaling effective menopause support packages tailored to the needs of autistic women.

## Data Availability

The original contributions presented in the study are included in the article/Supplementary Material, further inquiries can be directed to the corresponding author.
